# Eating behaviors, depression, and anxiety levels of pre bariatric surgery patients with obesity comorbid with or without Attention deficit and hyperactivity disorder: ADHD or Major Depression? Which is more related with eating behaviors?

**DOI:** 10.1002/brb3.1915

**Published:** 2020-10-28

**Authors:** Ebru Şahan, Meliha Zengin Eroğlu, Sencan Sertçelik

**Affiliations:** ^1^ Department of Psychiatry Marmara University İstanbul Turkey; ^2^ Department of Psychiatry Haydarpaşa Numune Training and Research Hospital İstanbul Turkey

**Keywords:** bariatric surgery, binge eating, disinhibition of eating, emotional eating, susceptibility to hunger

## Abstract

**Objective:**

A high rate of attention deficit and hyperactivity disorder **(**ADHD) has been reported in patients undergoing obesity treatment. It is controversial whether ADHD solely or its comorbid disorders account for eating behaviors associated with obesity.

**Methods:**

After presurgery psychiatric assessment, 100 severely obese patients (50 with ADHD and 50 without ADHD) were administered Adult Attention Deficit Hyperactivity Disorder Self‐Report Scale(ASRS), Wender Utah Rating Scale(WURS), Three‐Factor Eating Questionnaire(TFEQ), and Beck Depression Inventory(BDI) and Beck Anxiety Inventory(BAI).

**Results:**

Patients with obesity and ADHD had significantly greater emotional eating, susceptibility to hunger, depression, and anxiety but less restraint of eating scores than those without ADHD. Disinhibition of eating scores and presence of Binge Eating Disorder(BED) did not differ significantly between ADHD and non‐ADHD groups. Obese patients with major depression had significantly higher ASRS, WURS, TFEQ, BAI scores, disinhibition of eating control, emotional eating, susceptibility to hunger, and diagnosis of BED than nondepressed ones.

**Conclusions:**

Major depression and anxiety disorder have associations with disinhibition of eating control, emotional eating, susceptibility to hunger and BED, ADHD. Disinhibition of eating and BED did not differ according to the presence of ADHD; thus, depression was associated with eating control on more constructs than ADHD in our study.

## Graphical abstract

Major Depression has associations with almost all disordered eating behaviors including disinhibition of eating control, emotional eating, susceptibility to hunger and BED, ADHD and anxiety disorder. Disinhibition of eating and BED did not differ according to the presence of ADHD in our sample; thus, depression seems to be associated with eating control on more constructs than ADHD.

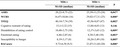

## 1 INTRODUCTION

Mood disorders, anxiety disorders, and eating disorders were reported to be the most prevalent categories of psychiatric disorders in obese patients (Lin et al., 2013). Previous studies have revealed high rates of attention deficit and hyperactivity disorder **(**ADHD) in adults receiving obesity treatment (Cortese & Vincenzi, 2012). The frequency of ADHD was found to be 27.4% in the study of the Altfas (2002), reaching up to 42.6%, especially in the group with a body mass index of 40 or above. Patients with ADHD are documented to have significantly higher BMI than patients without ADHD (Blum et al., 2000).

Evaluation of eating patterns, eating disorders, and problematic eating behaviors such as night eating syndrome, emotional eating, loss of control while eating are important in understanding how one develops severe obesity. Some possible pathological mechanisms are postulated between ADHD and obesity. First, impulsivity, deficits in inhibitory control, and executive functions associated with ADHD may lead to disordered eating behaviors (Cortese & Vincenzi, 2012). Second, the consumption of highly palatable food can activate dopamine in the common reward pathway and serve as self‐medication. Since high‐calorie food is readily available and legal, the treatment success of obesity is lower in the presence of ADHD (Davis et al., 2006). In a community sample, ADHD predicted binging and/or purging, but not the restrictive eating behaviors (Bleck & DeBate, 2013).

Nearly 90% of adults with ADHD have comorbid psychiatric disorders which may hide ADHD symptoms (Nutt et al., 2007). Anxiety, depression, and other mood disorders are frequently associated with ADHD (Corbisiero et al., 2013; Gillberg et al., 2004; Lin et al., 2015; Miller et al., 2007; Sobanski et al., 2007), and these may be both cause and consequence of obesity.

Obesity was related to a 25% increase in the likelihood of mood and anxiety disorders in an adult population (Simon et al., 2006). The co‐occurrence of depression and obesity is linked with challenges during the course of obesity surgery, increased disability, and burden (Mansur et al., 2015). Evidence from many trials concludes that depressed patients lose less weight than nondepressed cases and also have worse long‐term weight‐loss sustainment (de Zwaan, et al., 2011a, 2011b; Legenbauer et al., 2009; Ohsiek & Williams, 2011; Pauli‐Pott et al., 2013). Levels of anxiety and depression were found to be quite high in bariatric surgery candidates with food addiction (Eroğlu, 2019). There is only a few, mixed prospective evidence about the impact of obesity on anxiety disorder (Gariepy et al., 2010).

Past study samples of ADHD and obesity relationship included children, adolescents, mostly adult women, few men, or sometimes surgical obese patients. Binge eating was the most assessed eating pathology. Some studies have investigated direct and indirect pathways (Kaisari et al., 2018), mediators, and confounding factors between ADHD, comorbid disorders, and obesity. Some found the depression or binge eating as mediators of the relationship between ADHD and obesity (Cortese et al., 2013; de Zwaan, et al., 2011a, 2011b; Pagoto et al., 2009; Tong et al., 2017). However, the results of the individual contribution of each psychiatric disorder to obesity are still controversial. We aimed to group obese patients preparing for weight‐loss surgery as those with and without ADHD and then to assess eating patterns like restraint of eating, emotional eating and susceptibility to hunger, depression, anxiety, and other contributing factors to obesity in these groups. Screening and effectively managing psychiatric comorbidities might have the potential to reduce not only personal and social adverse outcomes but also to increase the success of obesity treatments and the quality of life of these patients (Cortese et al., 2013).

## 2 METHODS

This study is approved by Bezmialem Vakif University Noninvasive Research Ethics Committee on 20.07.2018 with number 54022451–050.05.04‐.

### 2.1 Inclusion criteria

1. To be over 17 and less than 67 years of age
2. Being super‐obese (Grade IV obesity, BMI > 50), morbidly obese (Grade III obesity, BMI > 40), or severely obese (Grade II obesity, BMI 35–40 with at least one comorbid medical disease)
3. Completion of pre bariatric surgery psychiatric consultation
4. Willing to participate in the study and giving written informed consent

### 2.2 Exclusion criteria

1. To be under the age of 17 or older than 67
2. Illiteracy
3. Having a visual disability
4. A diagnosis of psychosis, mental retardation, or dementia
5. A diagnosis of alcohol and/or substance use disorder
6. Patients previously diagnosed with ADHD and using stimulants for the treatment

**Figure 1:**
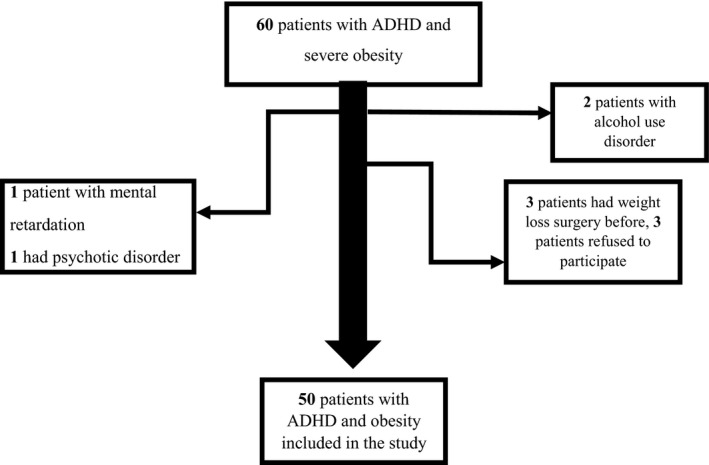
Flipchart of excluded cases with ADHD and severe obesity

Grade III and IV obese patients and Grade II obese patients with at least one comorbid medical disease who admitted to study centers as bariatric surgical candidates were evaluated routinely for ADHD at presurgical psychiatric interviews. Information about the study was given to patients. Two participants with alcohol use disorder, 1 with mental retardation, and 1 with a psychotic disorder were excluded from the study. 3 surgery applicants for the second time (gastric bypass surgery) were excluded, and 3 patients did not participate voluntarily (Figure 1). After exclusion, 50 patients with obesity and ADHD who gave written informed consent to participate were consecutively included. The control group was formed from the same sample with 50 volunteers without ADHD. 52 of participants were from admissions to Bezmialem Vakif University and 48 to Haydarpaşa Numune Training and Research Hospital.

Structured Clinical Diagnostic Interview (SCID‐V) was used to determine the diagnoses of ADHD, BED, major depressive disorder (MDD), and anxiety disorders (AD) according to DSM‐5. Adult Attention Deficit Hyperactivity Disorder Self‐Report Scale (ASRS), Wender Utah Rating Scale (WURS), Three‐Factor Eating Questionnaire (TFEQ) and Beck Depression Inventory (BDI), and Beck Anxiety Inventory (BAI) were administered.

### 2.3 Data collection tools and features

#### 2.3.1 Structured Clinical Diagnostic Interview (SCID‐V)

Structured Clinical Interview for DSM‐5‐ClinicianVersion is a semi‐structured interview developed for DSM‐V diagnoses (First, 2015) and is used by trained interviewers. It consists of ten modules. 32 diagnostic categories have detailed diagnostic criteria, while 17 diagnostic categories have only investigative questions. Turkish reliability and validity study was conducted by Bayad et al. (2018).

#### 2.3.2 Adult attention deficit hyperactivity disorder self‐report scale

Validity and reliability study of the Turkish version of the Adult Attention Deficit Hyperactivity Disorder Self‐Report Scale (ASRS) was conducted in 2009 by Dogan et al. (2009). Cronbach's alpha score for internal consistency was 0.88, and for test–retest reliability, it was 0.81. It was shown to be a valid and reliable tool, and it has been used as an aid in screening for adult attention deficit hyperactivity disorder.

### 2.4 Wender Utah rating scale (WURS)

Wender Utah Rating Scale (WURS) is a scale that can help to diagnose Attention Deficit and Hyperactivity Disorder (ADHD) in adults. WURS is a five‐point Likert‐type self‐report scale in which each item is rated 0–4 (0 = none, 4 = extreme). The cutoff score was 36. When this cutoff point was taken, the sensitivity was 82.5% and the specificity was 90.8%. The validity and reliability study of the Turkish 25‐item version was done in 2005. For the internal consistency analysis of WURS, Cronbach alpha coefficient was 0.93 for the whole scale. Item‐total score correlations were between 0.60 and 0.75. One month test–retest reliability was found to be 0.81. It was shown to be a valid and reliable scale that can help clinicians to diagnose ADHD in adults (Öncü et al., 2005).

### 2.5 Three‐factor eating questionnaire (TFEQ)

TFEQ measures eating behavior with three sub‐factors called cognitive restraint of eating, disinhibition of eating control, and emotional eating (Bond et al., 2001). Higher scores denote higher levels of each factor, respectively. The validity and reliability study of the Turkish version of the 3‐Factor Eating Scale (TFEQ) was conducted by Kıraç et al. (2015). Items 1, 7, 13, 14, and 17 measure uncontrolled eating; 3, 6, and 10 measure emotional eating levels; items 2, 11, 12, 15, 16, and 18. measure cognitive restraint of eating. Factor analysis and varimax rotation gave the other factor, susceptibility to hunger. Items 4, 5, 8, and 9 measure the sensitivity to hunger. The internal consistency of TFEQ has been tested with Cronbach's alpha reliability coefficient and the score for internal consistency was 0.70 and the test–retest reliability for all four factors was above 0.92. The scale was found suitable for Turkish culture and determined as a valid and reliable instrument to verify eating behaviors (Kıraç et al., 2015).

### 2.6 Beck depression inventory (BDI)

BDI is a 21 item self‐report scale developed by Beck et al. (1961). Items in the scale are rated from 0 to 3 in increasing order of severity. Item scores are summed, and the total score can range from 0 to 63. Higher scores correlate with more severe depression. The validity and reliability of the BDI in the Turkish population have been studied by Hisli et al. (1989). Cronbach's alpha was 0.92 in the present study.

### 2.7 Beck anxiety inventory (BAI)

Anxiety is measured using the 21‐item self‐report BAI (Beck et al., 1988). Each item is scored from 0 to 3 according to severity. Item scores are totaled, and the total score correlates with anxiety level. Ulusoy et al. (1998) studied the Turkish validity and reliability of the BAI. Cronbach's alpha was found to be 0.92.

### 2.8 Statistical analysis

IBM Statistical Package for Social Sciences for Windows (SPSS) version 22.0 (SPSS, Chicago, Illinois) program was used to evaluate findings in this study. Categorical variables are reported as numbers and percentages. Continuous variables were checked for the normal distribution assumption using the Shapiro–Wilk test. Kruskal–Wallis test was used for comparison of nonparametric quantitative data. Mann–Whitney U test was used to compare nonparametric variables between two groups. Fisher's exact test and Continuity (Yates) Correction were used to compare qualitative data. Spearman's rho test was used to investigate the relationships between numerical variables. Significance was evaluated at *p* < .05.

## 3 RESULTS

The mean BMI of non‐ADHD obese patients was 44.81 ± 4.88, and the mean BMI of ADHD obese was 45.35 ± 5.21. The mean age of non‐ADHD obese patients was 34.46 ± 10.54, and the mean age of ADHD patients with obesity was 35.59 ± 12.12. Sociodemographic characteristics and scale scores of the participants are shown in Table 1.

**Table 1:** Sociodemographic characteristics and scale scores of the participants

		Obese non‐ADHD	Obese ADHD
		Ort ± SS	Ort ± SS
Sex *_n(%)_*			
Male		17 (%34)	11 (%22)
Female		33 (%66)	39 (%78)
Marital Status*_n(%)_*			
Single		21 (%42)	20 (%40,8)
Married		29 (%58)	28 (%57,1)
Divorced		0 (%0)	1 (%2)
Educational Status*_n(%)_*			
Primary school		18 (%36)	19 (%38,8)
High school		19 (%38)	15 (%30,6)
University		13 (%26)	15 (%30,6)
Medical Comorbidity *_n(%)_*			
No		7 (%14)	13 (%26,5)
Yes		43 (%86)	36 (%73,5)
Psychiatric comorbidity *_n(%)_*			
No		39 (%78)	34 (%69,4)
Yes		11 (%22)	15 (%30,6)
Family history of psychiatric disorders *_n(%)_*			
No		44 (%88)	41 (%83,7)
Yes		6 (%12)	8 (%16,3)
Alcohol and substance use *_n(%)_*			
No		43 (%86)	44 (%89,8)
Yes		7 (%14)	5 (%10,2)
Smoking *_n(%)_*			
No		31 (%62)	27 (%55,1)
Yes		19 (%38)	22 (%44,9)

Abbreviation: ADHD, Adult Attention Deficit Hyperactivity Disorder.

The rate of patients with obesity for at least one psychiatric disorder was found as 22.0%, two psychiatric disorders 14.0%, and three psychiatric disorders 7%. Psychiatric evaluations were made by a psychiatrist based on SCID‐5. Distribution of psychiatric disorders was major depression 20.0%, generalized anxiety disorder 12%, social phobia 5.0%, panic disorder 5.0%, obsessive–compulsive disorder 2.5%, and post‐traumatic stress disorder 1%. Bulimia nervosa and anorexia nervosa were not found.

Women participants of our study had higher scores on emotional eating (*p* = .017), susceptibility to hunger (*p* = .040), and BAI scores (*p* = .011) than men. Participants who had psychiatric treatment in the past or receiving psychiatric treatment at the time of assessment had higher disinhibition of eating control (*p* = .044) and lower cognitive restraint of eating (*p* = .003) scores than those without a psychiatric disorder in the whole sample.

The percentage of Binge Eating Disorder in obese ADHD participants was 16.3%, and the percentage of BED in non‐ADHD obese participants was 6% (Continuity Yates correction *p* = .189). Table 2 shows the comparisons of eating behaviors, BDI, and BAI scores between ADHD and non‐ADHD obese participants.

**Table 2:** Evaluation of the TFEQ score, BDI and BAI scores in obese patients with or without ADHD

	Obese non‐ADHD	Obese ADHD	*p*
	*M* ± *SD* (median)	*M* ± *SD* (median)	
TFEQ	41,7 ± 8,46 (41)	43,66 ± 8,09 (44,5)	.200
Cognitive restraint of eating	15,36 ± 3,74 (16)	13,64 ± 3,45 (14)	.013*
Disinhibition of eating control	11,02 ± 4,06 (11)	11,69 ± 3,51 (11)	.387
Emotional eating	6,7 ± 3,32 (6)	8,22 ± 3,11 (8)	.020*
Susceptibility to hunger	8,42 ± 3,35 (8)	9,91 ± 3,44 (10)	.037*
BDI score	13,92 ± 8,61 (12,5)	18,8 ± 9,85 (16)	.015*
BAI score	10,38 ± 7,53 (9)	19,87 ± 11,9 (17,5)	.000*

Abbreviations: ADHD, Adult Attention Deficit Hyperactivity Disorder; BAI, Beck Anxiety InventoryBDI, Beck Depression Inventory; TFEQ, Three‐Factor Eating Questionnaire.

*Mann–Whitney U test*

* *p* < .05

The percentage of Binge Eating Disorder in obese participants with major depression was %21.7, and the percentage of BED in obese participants without depression was %1.9 (Continuity Yates correction *p* = .005). Table 3 shows the comparisons of ASRS, WURS, TFEQ, and BAI scores among obese patients with and without MD.

**Table 3:** Assessment of ASRS, WURS, TFEQ, and BAI scores among obese patients with and without MD

	MD(‐)	MD(+)	*p*
	*M* ± *SD* (median)	*M* ± *SD* (median)	
ASRS	20.22 ± 8.75 (22)	25.39 ± 11.55 (24)	.047*
WURS	16.47 ± 10.06 (16)	29.02 ± 17.31 (25)	.000*
TFEQ	40.14 ± 7.74 (39)	45.56 ± 8.07 (47)	.002*
Cognitive restraint of eating	15.1 ± 3.32 (15)	13.88 ± 4.03 (13)	.146
Disinhibition of eating control	10.48 ± 3.75 (10)	12.37 ± 3.65 (12)	.009*
Emotional eating	6.46 ± 2.85 (6)	8.58 ± 3.46 (10)	.003*
to hunger	8.19 ± 3.17 (8)	10.26 ± 3.49 (10)	.005*
BAI score	9.73 ± 6.58 (9,5)	21.07 ± 11.84 (20)	.000*

Abbreviations: *ASRS, Adult Attention Deficit Hyperactivity Disorder Self‐Report Scale; BAI, Beck Anxiety Inventory; Mann–Whitney U test; MD, Major Depression; TFEQ, Three‐Factor Eating Questionnaire;WURS, Wender Utah Rating Scale*.

* *p* < .05

The percentage of Binge Eating Disorder in obese participants with an anxiety disorder was 18.2%, and the percentage of BED in obese participants without depression was 6.5%. (Continuity Yates correction *p* = 0.091).

Table 4 shows the comparisons of ASRS, WURS, TFEQ, and BDI scores among obese patients with and without Anxiety Disorder.

**Table 4:** ASRS, WURS, TFEQ, BDI scores and the presence of BED between obese patients with and without an anxiety disorder

	No anxiety disorder	Anxiety disorder	*p*
	*M* ± *SD* (median)	*M* ± *SD* (median)	
ASRS	19.02 ± 8.57 (18,5)	28.47 ± 11.11 (27)	.000*
WURS	18.57 ± 12.49 (17)	28.67 ± 17.78 (25)	.005*
TFEQ	40.9 ± 8.01 (39)	45.84 ± 8.25 (47)	.007*
Cognitive restraint of eating	14.78 ± 3.66 (14,5)	13.94 ± 3.7 (14)	.218
Disinhibition of eating control	10.8 ± 3.84 (10)	12.34 ± 3,66 (12)	.047*
Emotional eating	6.72 ± 3.06 (6)	8.84 ± 3,33 (10)	.005*
Susceptibility to hunger	8.5 ± 3.25 (8)	10.41 ± 3.55 (10)	.012*
BDI score	12.42 ± 7.45 (11,5)	23.24 ± 8.8 (24)	.000*

Abbreviations: *ASRS, Adult Attention Deficit Hyperactivity Disorder Self‐Report Scale; BDI, Beck Depression Inventory; Mann–Whitney U testTFEQ, Three‐Factor Eating Questionnaire;WURS, Wender Utah Rating Scale*.

* *p* < .05.

Table 5 shows how do all these scale scores correlate with each other.

**Table 5:** Correlations of scales

	1	2	3	4	5	6	7	8	9
1. ASRS									
2. WURS	0.502**								
3. TFEQ	0.169	0.143							
4. TFEQ Cognitive Restraint	−0.294**	−0.105	−0.032						
5. TFEQ Disinhibition	0.128	0.144	0.707**	−0.410**					
6. TFEQ Emotional Eating	0.312**	0.183	0.718**	−0.257*	0.373**				
7. TFEQ Hunger	0.231*	0.146	0.809**	−0.359**	0.643**	0.576**			
8. BDI	0.372**	0.490**	0.422**	−0.172	0.352**	0.397**	0.398**		
9. BAI	0.507**	0.369**	0.412**	−0.105	0.273**	0.329**	0.394**	0.626**	

ASRS, Adult Attention Deficit Hyperactivity Disorder Self‐Report Scale; BAI, Beck Anxiety Inventory; BDI, Beck Depression Inventory; TFEQ, Three‐Factor Eating Questionnaire; WURS, Wender Utah Rating Scale.

*p *< .05

** *p *< .01

## 4 DISCUSSION

Patients with obesity and ADHD had significantly greater emotional eating, susceptibility to hunger, depression, and anxiety but less restraint of eating scores than those without ADHD. Disinhibition of eating scores and the presence of Binge Eating Disorder(BED) did not differ significantly between ADHD and non‐ADHD groups. Obese patients with major depression or anxiety disorder had significantly higher ASRS, WURS, TFEQ, BAI scores, disinhibition of eating control, emotional eating, susceptibility to hunger, and diagnosis of BED than nondepressed or nonanxious ones in our study.

Our study revealed that obese ADHD patients—both men and women—were less successful in cognitive restraint of eating. In ADHD adults, the readiness to act rashly without worrying about future outcomes can bring on unrestrained eating which can be one of the pathways in the development of obesity. In a study of 223 obese women, those scoring higher on restraint at baseline had lower body weights. During weight‐loss treatment, as restraint score increased, disinhibition and hunger susceptibility decreased. Besides, patients who increased restraint scores during treatment lost more weight (Foster et al., 1998). Most accept that ADHD is connected with deficits in inhibitory control, which is observed behaviorally as poor planning for the future and the inability to monitor one's behavior efficaciously (Cepeda et al., 2000; Clark et al., 2000). Appropriate eating behaviors for weight management necessitate significant planning, organization, and self‐regulation—compelling strategies for people with inadequate inhibitory control (Davis et al., 2006). In a 16‐week weight‐loss program perceived self‐efficacy to control eating was lower in individuals with ADHD than controls (Pagoto et al., 2010). Deficient inhibitory control could precede over‐consumption by the recklessness of daily caloric intake.

We found higher disinhibition of eating scores in our ADHD group but it did not show a significant statistical difference from patients without ADHD. This was an unexpected finding and might be related to the sample characteristics—severely obese patients being recruited from very specific hospital settings. ADHD may increase the risk of obesity in general population as stated in the previous literature; however, obesity has several other causes. Especially, ADHD might not represent a significant risk factor for disinhibition of eating in patients with severe obesity according to our findings.

ADHD obesity relationship has many different aspects and other eating behaviors like impulsivity related to eating, eating fast, and eating when not hungry might be as important as disinhibition (Smith et al., 1999). Besides, patients with ADHD may be relatively careless for internal hunger and satiety signals (Fleming et al., 2005).

Emotional eating can be delineated as eating to cope with a range of negative feelings (Tanofsky‐Kraff et al., 2007). Women had higher emotional eating scores in our study which confirms the sex difference result of emotional eating in a large cohort study. They included participants from all BMI categories and both dieters and those never dieted but we included only severe obese subjects who have all dieted before and still found emotional eating is more prominent in women (Péneau et al., 2013). Our study and most earlier studies showed eating food in response to emotional distress correlated positively with ADHD in obese adults (Alfonsson et al., 2012, 2013; Pagoto et al., 2010). It is imaginable that to handle frustrations and negative affect resulting from the consequences of attention problems (eg poor work accomplishment) and/or impulsive reactions at the cost of planned objectives, people with ADHD could be into disordered eating (Kaisari et al., 2018). Notwithstanding, in overweight/obese children and adolescents Pauli‐Pott et al. (2013) found no significant difference in emotional eating between groups with clinical and/or subclinical ADHD symptoms and without ADHD symptoms. They included only 17 children–adolescents and included both subclinical and clinical ADHD though we included 50 adults and excluded subclinical ADHD. Dissimilarities of sample size and characteristics may have affected the difference in emotional eating.

Susceptibility to hunger is the raised responsivity to noticed body symptoms that indicate the need for food (Dempsey et al., 2011). We found higher susceptibility to hunger scores in the ADHD group as the study by Dempsey et al. which used path analysis. Patients with ADHD choose immediate even if the latter will bring greater rewards (Banaschewski et al., 2005). For instance, avoiding a delay may enhance the disposition to consume fast food instead of homemade meals which need cooking and more time to prepare (Dempsey et al., 2011). Women were more susceptible to hunger than men. Dempsey's study (2011) did not compare hunger in ADHD according to gender but in a study about cerebral responses to food‐related stimuli women had stronger hunger effects, reacted more strongly to external food‐related stimuli than men after fasting (Uher et al., 2006).

The findings of this study show that pre bariatric obese patients with ADHD had significantly greater emotional eating, susceptibility to hunger scores than obese patients without ADHD. They were significantly less capable of cognitive restraint of eating. In a review of twelve studies, behaviors related to overeating, including emotional eating, hedonic eating, external eating, eating as a result of increased susceptibility to hunger, binge and disinhibited eating had significant positive associations with ADHD in 10 of the 12 studies (Kaisari et al., 2017). And our study affirmed that emotional eating and susceptibility to hunger were significantly associated with ADHD; however, associations of binge eating and disinhibited eating to ADHD were not significant.

In the studies with adult healthy women and men, ADHD symptoms correlated positively with aspects of overeating and ultimately correlated with higher BMI (Davis et al., 2006; Strimas et al., 2008). However, our study included only severely obese patients at baseline so we did not compare BMI with eating behaviors in our ADHD sample.

The prevalence of self‐reported BED in bariatric surgery candidates was 15,4% in the study by Mitchell, et al. (2015), and the frequency of BED via a psychiatric interview in our whole group was a near estimate 11%. It is known that self‐regulation is a well‐documented challenge for ADHD patients and can lead to chronic overeating or binge eating patterns (Quinn, 2008). The generalization that ADHD predicts binging and/or purging behavior but not restrictive behaviors made us predict that BN and BED should always be with ADHD. Here, we suggested that BED is not associated with compensatory purging; therefore, it should cause more weight gain and should be more common in severely obese patients (Bleck & DeBate, 2013). The ADHD group had a higher BED ratio than the non‐ADHD group, but the difference between the groups did not reach statistical significance (*p* = .189). This was surprising and not consistent with some of the studies in the literature. The number of patients with BED was low (*n* = 11, 8 with ADHD and 3, not ADHD) in our sample which might have concealed a significant difference. It is not clear how much relevant is BED for the obesity–ADHD relationship and which mediators are involved. de Zwaan, et al. (2011a2011a, 2011b) claims that the relationship between obesity and adult ADHD was not fully accounted for with binge eating.

From the opposite side, a previous study exploring lifetime and past 12‐month binge eaters in both sexes for ADHD found a significantly higher prevalence of ADHD in binge eaters. Following the beginning age of ADHD is earlier than the Eating Disorders (ED), they argued that ADHD can be an eminent risk factor for binge eating and related EDs (Brewerton & Duncan, 2016). Fernández‐Aranda (2013) implied a positive relationship between the frequency of binge eating episodes and ADHD; thus, ADHD was queried as a secondary psychopathological estimate of binging severity.

Some other studies did not find an association between binge eating and ADHD like ours. In a case–control study, Davis et al. (2009) predicted that ADHD symptoms would be more severe in binge eaters compared to obese subjects without binge eating. On the contrary, they found that ADHD symptoms were increased in obese adults, with or without binge eating and the two groups were not statistically different.

The number of patients with BED was low in our sample; therefore, it would not be statistically appropriate to compare binge eaters with nonbinge eaters according to psychopathology. In a study with 90 extremely obese individuals, the patients who have reported regular binge eating were not different from those who did not report regular binge eating concerning BMI, age, sex, reactive temperament, and the incidence of adult ADHD. On the other hand, after regression analysis, regular binge eating was associated with eating concerns and reduced effortful control (Muller et al., 2012). The inattentive symptoms of ADHD were directly, negative mood, and lack of awareness and reliance on internal hunger/satiety signals indirectly related to binge/disinhibited eating (Kaisari et al., 2018). Emotion regulation deficits may be present in the loss of control of eating (Hartmann et al., 2013). In the study by Ziobrowski et al. (2017), although lifetime ADHD was strongly and significantly associated with lifetime BN and BED after adjustment for demographic variables and psychiatric comorbidities, only BN which remained significant. They suggested that the ascertained former relationship between ADHD and ED may be at least partly due to additional psychiatric disorders that are associated with both ADHD and EDs. This view may also explain why we could not find a significant difference in BED between patients with or without ADHD but we found BED significantly higher in patients with major depression.

Before surgical treatment, a substantial proportion of obese patients report disordered eating behaviors, most implying mental health problems (Mitchell, et al., 2015). Similarly, from 100 morbidly obese patients who had obesity surgery, 40% with a psychiatric disorder had a more disordered eating pattern (predominantly binge eating and disinhibition) than 60% without a psychiatric disorder (Guisado et al., 2001). In our study, participants who had psychiatric treatment in the past or receiving psychiatric treatment at the time of assessment had higher disinhibition of eating control and lower cognitive restraint of eating scores than those without a lifetime psychiatric disorder in the whole sample. We could not exactly know if this was related to psychiatric illness or treatment because of the cross‐sectional design and small number of patients under treatment (*n* = 7) and variability of medications, dosages, and durations in the treatments.

In a study using the Three‐Factor Eating Questionnaire, scores of the BED group on the disinhibition of eating and susceptibility to hunger subscales were significantly higher than the non‐BED group. The mean restraint score of the BED group on another scale was significantly higher than that of the non‐BED group (Goldfein et al., 1993). Evidence suggests that disinhibition scores correlate with binge eating severity (Foster et al., 1998). Disinhibition of eating scores was not significantly different between our ADHD and non‐ADHD patients with obesity; accordingly, the BED was not different between our ADHD and non‐ADHD groups.

Many studies are searching for predictors of BED but the results are inconsistent. In the study by Nazar et al., (2014) the presence of depressive symptoms, ADHD inattention symptoms, and trait‐impulsivity were the predictors of binge eating in obese patients in decreasing order of severity. Overall, active psychopharmacologic treatment, eating more frequently during the day, low self‐esteem, and higher depressive symptoms were proposed factors independently related to BED by Mitchell, et al. (2015) and our study identified the association of BED with life time or current psychiatric disorders and depression.

Severe obesity is strongly associated with depression even after adjusting for potential confounders. In an epidemiological study, patients with BMI ≥ 40 had a depression rate of 12.5% in the last month according to the Diagnostic Interview Schedule for DSM‐III (Onyike et al., 2003). Our descriptive analysis revealed the rate of self‐reported depression in obese patients as 28.8%. Patients with ADHD had significantly higher depression than obese patients without ADHD. Sobanski et al. (2006) observed that ADHD adults are impaired in daily living functions like fewer permanent partnerships, more divorces, less education, more often no professional training compared to healthy controls and these possibly have caused greater association with depression than controls.

Obese patients with major depression had significantly higher ASRS, WURS, TFEQ, BAI scores, disinhibition of eating control, emotional eating, susceptibility to hunger, and diagnosis of BED than nondepressed ones. Therefore, MD seems to be the most important psychopathology which has associations with almost all other disorders in our study. It is reasonable that mood changes related to depression, anxiety, and stress mediate the relationship between ADHD and the risk for problematic eating (Nutt et al., 2007). In a clinical setting, patients with MDD could reach 63%, whereas patients with BED could reach 55% of the weight loss of patients without these disorders, respectively. The effect of MDD on weight loss was not explained by BED or vice versa (Pagoto et al., 2007). Pagoto et al. reported that adult ADHD remained associated with overweight and obesity after controlling for major depressive disorder. Nonetheless, the association was no more statistically significant when controlling for BED in the past 12 months. Their conclusion that binge eating but not depression mediates partially the associations between ADHD and both overweight and obesity is contrary to our study (2009). Gruss et al. (2012) researched that adult ADHD was associated with greater depressive symptom severity and more psychotherapy contact in the past but not with BED or daytime sleepiness. Therefore, disordered eating may not be directly related to ADHD and might be mediated (at least in part) by comorbid anxiety and mood disorders (Quinn, 2008; Ziobrowski et al., 2017).

Many studies revealed that depression and anxiety are the most frequently encountered psychiatric comorbidities in ADHD patients (Friedrichs et al., 2012; Hodgkins et al., 2011; Rucklidge et al., 2016), especially in childhood‐onset ADHD social phobia may be prominent. The anxiety disorder rate was 27% in our study, and patients with ADHD had significantly higher BAI scores than patients without ADHD. Anxiety disorders were associated stronger with severe obesity (defined as a BMI⩾35) than with moderate obesity (BMI 30–35) (Gariepy et al., 2010). Petry et al. (2008) found adjusted ORs of past‐year and lifetime anxiety disorders increased as obesity severity increases. Obese patients with an anxiety disorder had significantly higher ASRS, WURS, TFEQ, BDI scores, disinhibition of eating control, emotional eating, and susceptibility to hunger than nonanxious ones. Anxiety was not related to BED and cognitive restraint of eating. One may guess that BED may not be a mediator for the anxiety–obesity relationship in our study.

Women had significantly greater BAI scores than men in our study. Anxiety may be related to emotional eating, especially in women. Barry et al. (2008) suggested that being overweight predicted an increased risk of social phobia and specific phobia for women but not for men. Obese women were at farther increased risk for social phobia which may be caused by harsh stigmatization and discrimination against female obese.

ADHD has many clinical implications for obesity treatment. First, the presence of ADHD has profound effects on both eating behaviors and comorbidities like depression and anxiety. Second, the treatment of ADHD with drugs like methylphenidate can help obese patients to lose weight. Third, ADHD has a dramatic impact on compliance with obesity treatment. Organizational difficulties, inattentiveness, forgetfulness, impulsivity, and procrastination in these patients may impede collaboration for postsurgical procedures (by not being able to attend to the control visits, not using the medicines regularly, poor planning for the order and time of meals, not controlling the amount and type of food to be eaten..). When working with obese ADHD patients, clients eating in response to emotions, and vulnerable to their perceptions of hunger should be supported in the acquisition of how to realize, preclude, and plan for these situations and psychiatrists also need to tailor the approach MD and AD (Dempsey et al., 2011).

This study was limited by small sample size, use of self‐report questionnaires, cross‐sectional study design which prevented causal interpretation of the relationships between parameters. The possible mediator or moderator variables like impulsivity were not covered. Our associations between eating behaviors, ADHD, MD, and AD should be seen as stringently hypothetical. In the future, there will be a need for prospective obesity studies, controlled for potentially confounding psychiatric conditions, designed with patients without comorbidities (e.g., pure major depression without ADHD, pure ADHD without MD even if it is hard to find them). Even so, this study contributes to the increasing body of knowledge about the relationship between obesity and eating behaviors, ADHD, MD, and anxiety, yet there is much that remains unknown.

## 5 Conclusions

Major Depression and anxiety disorder have associations with disinhibition of eating control, emotional eating, susceptibility to hunger, BED, and ADHD. Disinhibition of eating and BED did not differ according to the presence of ADHD; thus, depression and anxiety were associated with eating control on more constructs than ADHD in our study. The identification and effective management of psychiatric symptoms in obese individuals with treatment may alleviate more competent management of eating behaviors that yield obesity and may reduce the need for invasive and costly procedures for the treatment of obesity.

## Conflict of interest

We have no conflicts of interest to disclose. This study is not supported by any drug or other companies. No grants or fees were taken from any institution or firm.

## Author contributions

E.Ş. and M.Z.E. conceived of the presented idea and developed the theory. M.Z.E. and S.S. performed the computations and verified the analytical methods. E.Ş. and M.Z.E. wrote the manuscript in consultation with S.S who reviewed the literature. All authors discussed the results and contributed to the final manuscript.

### Peer Review

The peer review history for this article is available at https://publons.com/publon/10.1002/brb3.1915.

## Data Availability

The data that support the findings of this study are available on request from the corresponding author. The data are not publicly available due to privacy or ethical restrictions.
